# Case report: How I do it? Laparoscopic hepatectomy with transcatheter arterial ICG staining for hepatolithiasis review

**DOI:** 10.3389/fmed.2024.1470120

**Published:** 2024-11-05

**Authors:** Jingyi Xu, Xinye Qian, Wei Yang, Fei Yu, Yufan Yang, Wang Hu, Lu Gao, Shuang Wang, Liusheng Wu, Yutong Zhao, Lei Yang, Lin Zhang, Jun Yan

**Affiliations:** ^1^Center of Hepatobiliary Pancreatic Disease, Beijing Tsinghua Changgung Hospital, School of Clinical Medicine, Tsinghua University, Beijing, China; ^2^Department of General Surgery, The First People's Hospital of Shuangliu District, West China (Airport) Hospital Sichuan University, Chengdu, China; ^3^Department of Algology, Zhongshan Hospital, Fudan University, Shanghai, China; ^4^Department of Basic Medicine, Beijing Health Vocational College, Beijing, China

**Keywords:** vascular intervention, hepatolithiasis, laparoscopic hepatectomy, arterial ICG staining, case report

## Abstract

**Background:**

Hepatolithiasis is regarded as the presence of stones in the intrahepatic bile ducts. Recurrent inflammation of bile ducts can bring many bad effects. How to completely remove stones is still a challenge.

**Presentation:**

A 66-year-old male went to our hospital because of hepatolithiasis and choledocholithiasis. Selective hepatic arteriography and transcatheter arterial embolization followed by laparoscopic watershed hepatectomy under fluorescent navigation and laparoscopic common bile duct exploration on mass removal are performed.

**Clinical discussion:**

For patients who suffer from hepatolithiasis, the main treatment principles of the operation are to remove the stones, correct the stenosis, and prevent recurrence. The laparoscopic watershed hepatectomy under fluorescent navigation can provide important support for precision liver surgery, which can give some suggestions for future hepatectomy.

**Conclusion:**

Based on the three-dimensional (3D) visual watershed analysis, trans-arterial DSA positive fluorescence navigation has some advantages, which successfully overcome the shortcomings of the reverse staining method and the positive staining method.

## 1 Introduction

Hepatolithiasis is characterized by the formation of stones in the intrahepatic bile ducts. The chief complaint includes abdominal pain, fever, and jaundice (1). Although treatment options are not uniform, surgery is one of the most important interventions for hepatolithiasis as it could lower residual stone burden and lower recurrence rates when compared with pharmacologic treatment, lithotomy, and lithotripsy (2). However, limited resection of the liver is an independent risk factor for postoperative stone recurrence and long-term treatment failure because hepatolithiasis could affect an entire segment (3). One strategy to improve results is anatomical hepatectomy.

The development of laparoscopic fluorescence imaging technology has brought the possibility of true hepatectomy (4, 5). At present, there are two mainstream approaches. (1) Counterstaining method: after blocking the liver pedicle of the target liver segment, ICG is injected from the peripheral vein, and the reserved liver tissue is fluorescently stained, but the target liver watershed is not stained (6), thus showing the boundary of the target liver watershed. (2) Positive staining method: under the guidance of laparoscopic ultrasound, puncture the portal vein of the target liver segment and inject ICG to identify the boundary of the segment (7). The above methods have some disadvantages that are difficult to overcome: (1) The staining method requires dissecting the liver pedicle of the target segment outside the liver, which is not only difficult but also difficult to separate the liver pedicle of some segments deep inside the liver parenchyma, or there are multiple branches of the liver pedicle outside the liver. (2) The positive staining method puts forward very high requirements for endoscopic ultrasound-guided puncture technology. The thinner portal vein branches are difficult to locate and puncture, and the failure rate is high. Moreover, there might be multiple portal vein branches, so it is impossible to accurately control the injection dose and speed. (3) Due to the continuous scouring of blood flow in the blood vessels, there is an obvious problem of dye invasion into the adjacent liver tissue in both staining methods, that is, the non-target liver tissue also quickly appears fluorescent staining, resulting in the illegibility of the internal boundary of the liver parenchyma, which is a difficult problem to solve at present (8).

We present a novel procedure using positive arterial indocyanine green (ICG) staining under digital subtraction angiography (DSA) to visualize three targeted liver segments both onto and into the liver, which makes the anatomical hepatectomy easy to operate under fluorescence laparoscopy.

## 2 Method

### 2.1 Preparation

A 66-year-old male went to our hospital because of repeated epigastric pain for 5 years and aggravated it for 2 days. Five years ago, the patient experienced epigastric pain after eating greasy food. Other hospitals considered choledocholithiasis, and symptoms could be relieved after endoscopic retrograde cholangiopancreatography (ERCP). Then, the above symptoms of this patient existed multiple times and used ERCP in other hospitals many times (10 times in total). Abdominal pain symptoms of patients could be alleviated after treatments. Two days before visiting our hospital, the patient had epigastric pain with no obvious inducing factor. At the same time, the patient has chills and fever, and the maximum temperature is 38.7°C. Doctors in the emergency department considered patients had hepatolithiasis, choledocholithiasis, and acute cholangitis. The patient’s symptoms improved after anti-inflammation and antispasmodic treatment. The patient suffers from hypothyroidism, hypertension, and cerebrovascular disease, which make him take levothyroxine sodium tablets, irbesartan tablets, and aspirin enteric-coated tablets for a long time. He had accepted laparoscopic cholecystectomy in other hospitals. Meanwhile, he is allergic to moxifloxacin hydrochloride. After decades of smoking and drinking, he has quit smoking and drinking for 5 years. Patient denied genetic history. The patient had no special physical examination except for right upper abdominal tenderness.

**Figure 1:**
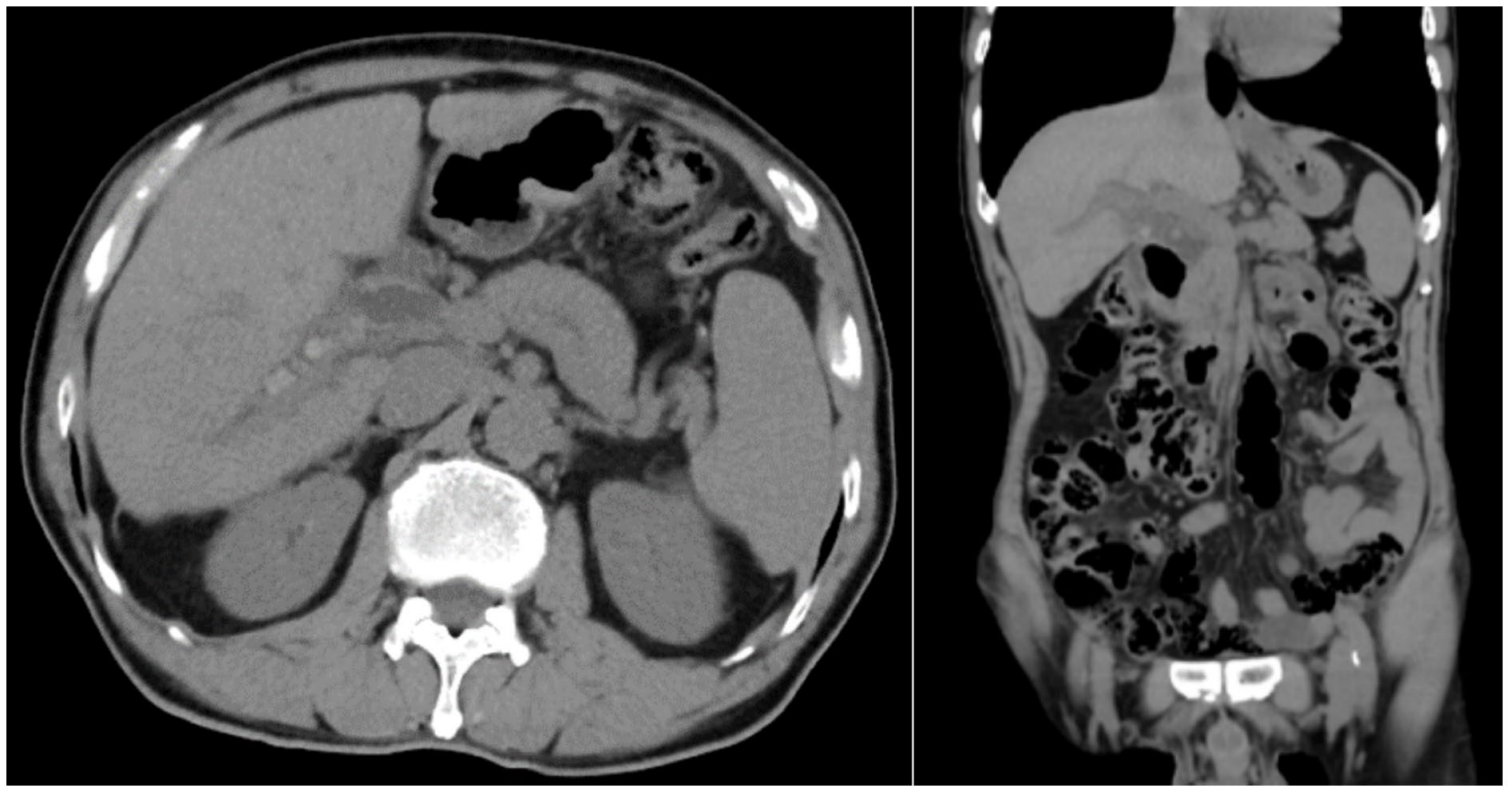
Preoperative CT result shows hepatolithiasis, dilatation of intrahepatic and extrahepatic bile ducts, and atrophic right posterior lobe of the liver.

Laboratory tests prompted an increase in C-reactive protein (CRP) levels to 25.59 mg/L (normal value is <5 mg/L) and procalcitonin (PCT) to 0.364 ng/mL (normal value is <0.05 ng/mL). Other blood tests had no obvious abnormality. In imageology, computed tomography (CT) revealed that there were multiple nodular high-density shadows in the porta hepatis and the internal bile duct of the right liver lobe, and the maximum diameter was approximately 11 mm. In addition, extrahepatic and intrahepatic bile duct dilation was shown, and the widest part of the common bile duct was approximately 17 mm. Moreover, in the lower segment of the common bile, we also found suspicious slightly high-density nodular shadows (Figure 1). Similar results were obtained on magnetic resonance cholangiopancreatography (MRCP). To sum up, the diagnosis of hepatolithiasis and choledocholithiasis was established.

Considering the long course of the patient’s illness and the risk of malignant transformation into cholangiocarcinoma (9), we performed an abdominal-enhanced CT examination on the patient. The results showed that no malignant tumor was found. At the same time, the patient’s tumor markers CA 19–9 (9.55 U/mL), CEA (1.85 ng/mL), AFP (2.15 ng/mL), and PIVKA II (23.56 mAU/mL) were normal, so we did not consider the patient to have malignant transformation.

Considering the poor effectiveness of conservative treatment in the patient’s internal medicine department, recurrent symptoms of cholangitis, inability to remove all stones through ERCP, and significant atrophy and loss of function in the right posterior lobe of the liver, there are surgical indications. After communication, the patient was planned to receive laparoscopic anatomical hepatectomy (S6 + S7) and laparoscopic common bile duct exploration (LCBDE).

### 2.2 Transcatheter arterial ICG staining

**Figure 2:**
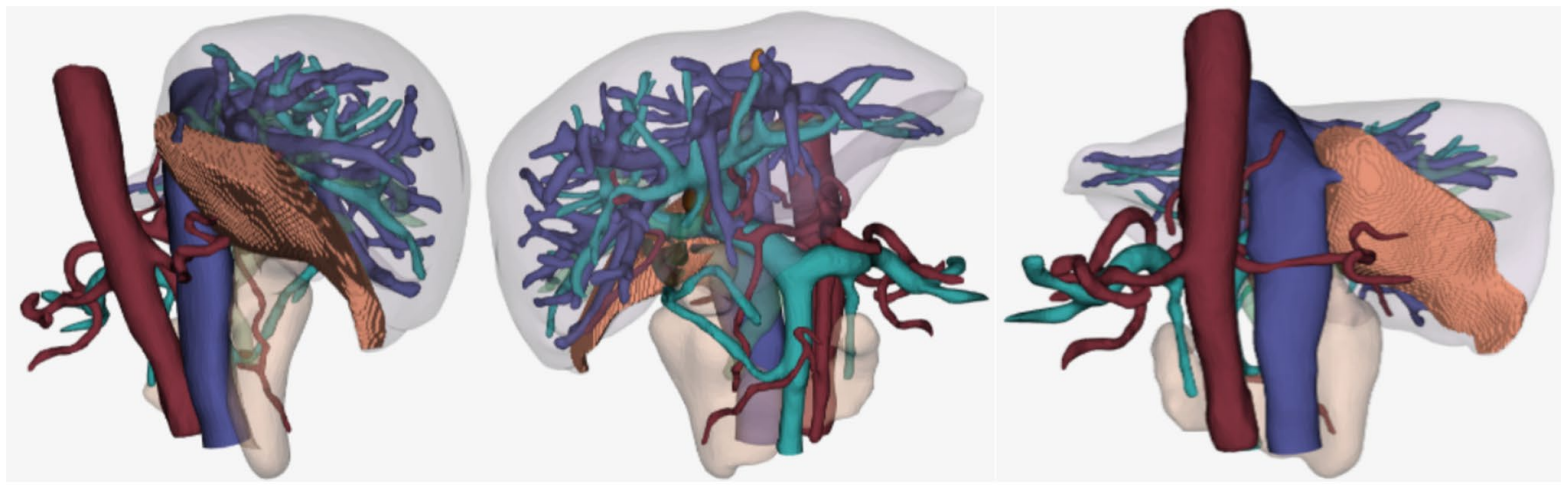
3D liver remodel, the shaded area is the watershed of the aimed hepatic artery. The watershed volume is 65.56 mL, the standard liver volume is 1281.71 mL, and the total liver volume is 1126.21 mL.

**Figure 3:**
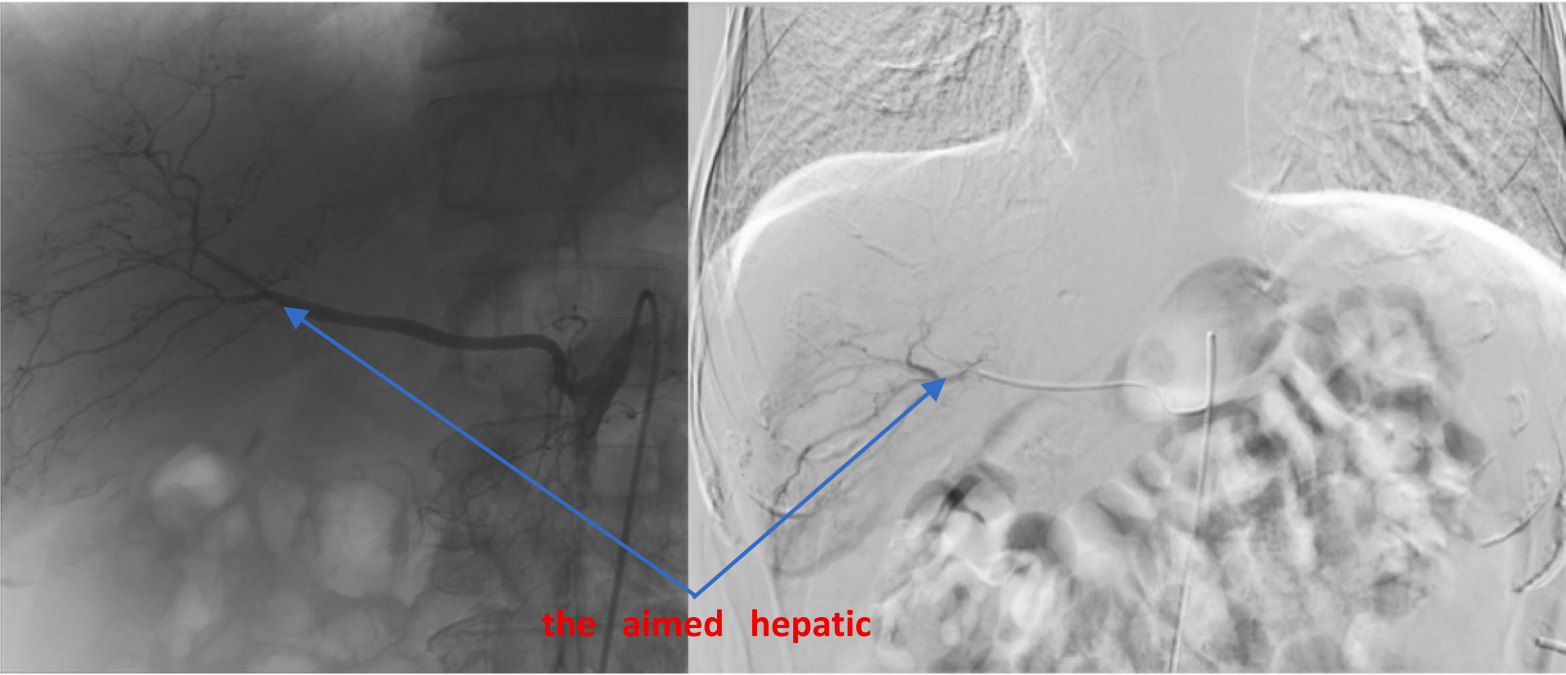
DSA, the aimed hepatic artery which guide wire entered.

**Figure 4:**
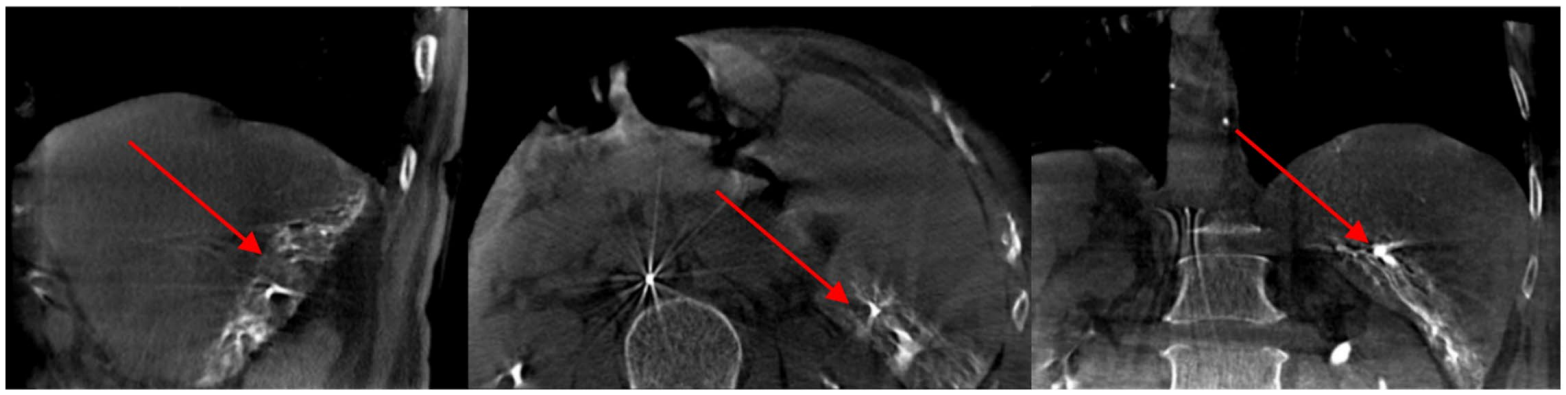
During the operation, the cone beam computed tomography (CBCT) result shows lipiodol was deposited in the right posterior lobe of the liver.

According to the preoperative CT-enhanced three-dimensional reconstruction (Figure 2) and surgical planning (targeted liver segment S6 + S7), the targeted hepatic artery was located first. A 2.4–2.8F microcatheter was used to superselect the arterial branches of the target liver watershed (Figure 3). Cone beam computed tomography (CBCT) was performed to confirm the consistency between the perfusion area of the selected hepatic artery and the preoperative liver watershed (Figure 4). Then, 3–5 mL of 1:1000 diluent ICG was injected, followed by 1:2 diluent lipiodol-based emulsion (embolization of the targeted hepatic artery). After general anesthesia, the surgery was started. The fluorescence mode of the laparoscopy was used to visualize the aimed liver watershed region. Laparoscopic watershed-oriented hepatic resection was performed by our team following the guidance of the fluorescent boundary.

### 2.3 Surgical resection under fluorescence laparoscopy

**Figure 5:**
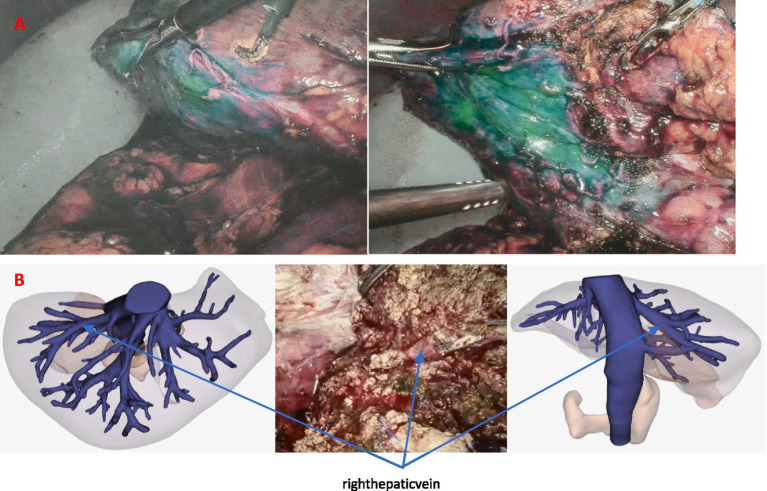
(A) Staining effect of the right posterior lobe (S6 + S7). (B) During the operation, the hepatic vein of the aimed watershed was highly accordant with 3D liver remodel.

Then, patients were sent to an operation room immediately. After the surgery was started, the fluorescence mode of the laparoscopy was used to visualize the aimed liver segment region. The staining effect of the right posterior lobe (S6 + S7) was fine (Figure 5). Under the guidance of fluorescence mode, porta hepatis was blocked intermittently, and the liver parenchyma was cut off by ultrasonic knife, using HomeLock (disposable organization closure clip, plastic clip) to clamp various branches of blood vessels and bile ducts in the liver section.

After the complete removal of S6 + S7, the common bile duct was cut open approximately 2 cm at the upper edge of the pancreas. Choledochoscopy revealed stones with a diameter of approximately 8 mm existing in the lower segment of the common bile, while the major duodenal papilla functioned well. A stone basket was used to remove common bile duct stones. However, the choledochoscope could not get into the stump of the right posterior hepatic duct. After flushing it with normal saline, five stones flowed out with a diameter of approximately 4 mm. No obvious residual stones were found in the common bile duct and intrahepatic bile duct; furthermore, the shape of the bile duct was normal under choledochoscope examination. The stump of the right posterior hepatic duct was closed by intermittent suture. T-tube (22F) was placed into the common bile duct and was sutured and fixed. We set drainage tubes on the liver section and foramen of Winslow.

## 3 Results

**Figure 6:**
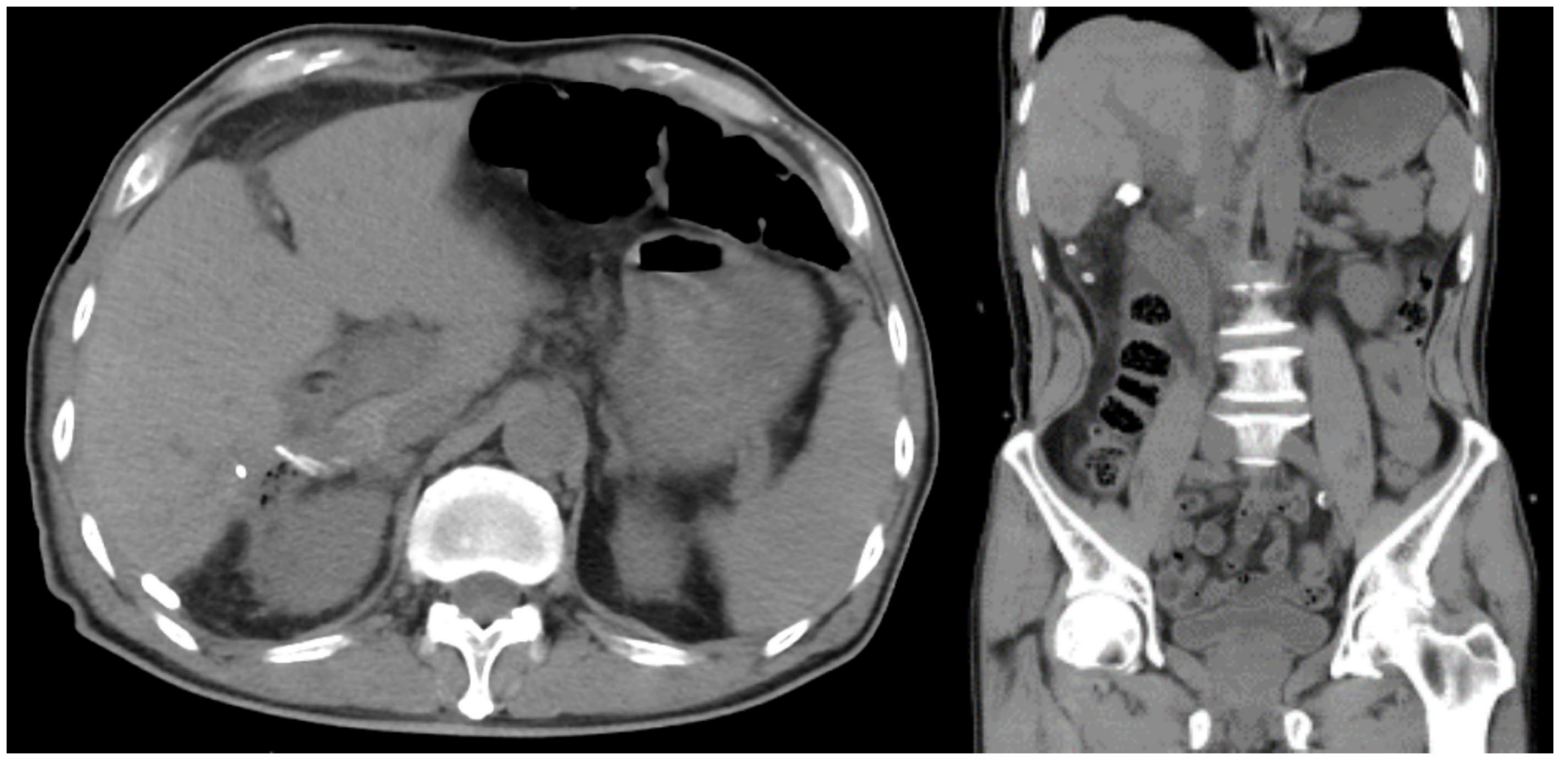
Nodular high-density shadows in the hilar bile duct are not shown on abdominal CT after the operation.

The operative time was 532 min, and the amount of bleeding was 200 mL. The pathologic diagnosis was intrahepatic bile duct dilation, acute and chronic cholangitis, and biliary duct stone. The patients had fever after 24 h of operation, and the maximum temperature was 38.6°C, which was considered to be related to the inflammation of the bile duct. After anti-infection treatment, the patient’s temperature gradually returned to normal, and there were no other complications after the operation. The abdominal drainage tube was removed on the 9th day after the operation, and the T-tube was closed on the 10th day. The patient recovered well and left the hospital on the 12th day after the operation. Following up after 2 years, the patient was safe and sound; abdominal CT was normal as well (Figure 6).

## 4 Discussion

As far as we know, this is the first case report of laparoscopic hepatectomy of the atrophic right posterior lobe using transcatheter arterial ICG staining in the world. This technology successfully overcomes the shortcomings of the existing staining methods, such as puncture difficulty and halo dyeing (10).

T-tubes are usually inserted during biliary surgery to ensure the smooth flow of bile and prevent biliary obstruction (11). After surgery, the management of T-tubes is crucial to ensuring the safety and recovery of patients and usually includes regular observation and cleaning (regularly checking the condition of the T-tube to ensure it is not blocked or dislodged and keeping it clean), monitoring bile outflow (observing the amount, color, and texture of bile outflow, evaluating the recovery of the biliary tract), and avoiding external pulling (ensuring that the T-tube does not detach or shift due to external pulling) (12). After evaluation, the patient resumed a normal diet 10 days after surgery without any discomfort, such as bloating or abdominal pain. To facilitate the digestion and absorption of food, we chose to clamp the T-tube to allow bile to be directly discharged into the intestine. Due to the patient’s regular follow-up CT and MRCP after surgery, all examinations did not indicate the presence of residual stones. Therefore, when we removed the T-tube 3 months after surgery, we did not perform transthoracic cholangiography to reduce invasive procedures (13).

Trans-arterial DSA positive fluorescence navigation has the following advantages: (1) The preoperative 3D visual surgical plan could be faithfully reproduced in the liver. (2) Whether on the surface of the liver or in the internal of the liver parenchyma, it could provide a clear, lasting, and full dimensional fluorescent navigation image. (3) It is unnecessary to deliberately look for anatomical marks in the liver. Corresponding anatomical marks can be naturally revealed along with the fluorescent image, which is highly matched with the 3D surgical plan. (4) It could be applied and repeated in other medical centers easily (14).

The application of this technology also provides important support for the concept of visualization, quantification, and controllability of precision liver surgery (15), which can give some suggestions for future hepatectomy.

## 5 Conclusion

Transcatheter arterial ICG staining is safe and feasible for visualization of 3D surgical planning both onto and into the liver, navigating laparoscopic liver segmentectomy precisely.

## Data Availability

The raw data supporting the conclusions of this article will be made available by the authors, without undue reservation.
